# Causal Effects between Gut Microbiome and Myalgic Encephalomyelitis/Chronic Fatigue Syndrome: A Two-Sample Mendelian Randomization Study

**DOI:** 10.3389/fmicb.2023.1190894

**Published:** 2023-07-06

**Authors:** Gang He, Yu Cao, Honghao Ma, Siran Guo, Wangzi Xu, Dai Wang, Yongquan Chen, Houzhao Wang

**Affiliations:** ^1^State Key laboratory of Molecular Vaccinology and Molecular Diagnostics. School of Public Health, Xiamen University, Xiamen, China; ^2^Yunnan Provincial Key Laboratory of Molecular Biology for Sinomedicine, Yunnan University of Traditional Chinese Medicine, Kunming, Yunnan, China; ^3^Department of Clinical Laboratory, Xiang’an Hospital of Xiamen University, Xiamen, Fujian, China

**Keywords:** gut microbiota, mendelian randomization, Myalgia Encephalomyelitis/Chronic Fatigue Syndrome, causal relationship, gut microbiome

## Abstract

**Background:**

Evidence from previous studies have implicated an important association between gut microbiota (GM) and Myalgic Encephalomyelitis/Chronic Fatigue Syndrome (ME/CFS), but whether there is a definite causal relationship between GM and ME/CFS has not been elucidated.

**Method:**

This study obtained instrumental variables of 211 GM taxa from the Genome Wide Association Study (GWAS), and mendelian randomization (MR) study was carried out to assess the effect of gut microbiota on ME/CFS risk from UK Biobank GWAS (2076 ME/CFS cases and 460,857 controls). Inverse variance weighted (IVW) was the primary method to analyze causality in this study, and a series of sensitivity analyses was performed to validate the robustness of the results.

**Results:**

The inverse variance weighted (IVW) method indicated that genus *Paraprevotella* (OR:1.001, 95%CI:1.000–1.003, value of *p* < 0.05) and *Ruminococca- ceae_UCG_014* (OR 1.003, 95% CI 1.000 to 1.005, value of *p* < 0.05) were positively associated with ME/CFS risk. Results from the weighted median method supported genus *Paraprevotella* (OR 1.003, 95% CI 1.000 to 1.005, value of *p* < 0.05) as a risk factor for ME/CFS.

**Conclusion:**

This study reveals a causal relationship between genus *paraprevotella*, genus *Ruminococcaceae_UCG_014* and ME/CFS, and our findings provide novel insights for further elucidating the developmental mechanisms mediated by the gut microbiota of ME/CFS.

## 1. Introduction

ME/CFS is a multisystemic disorder and an autoimmune disease, characterized by chronic and recurrent tiredness, which is frequently accompanied by sleep loss, neurocognitive impairment, irritable bowel syndrome, fever, headache, muscle weakness and soreness, and other symptoms (Sandler and Lloyd, 2020). Muscle fatigue occurs after mild physical exertion and is accompanied by impaired sensory, cognitive, and autonomic functions (Twisk, 2018). The underlying etiology or pathophysiology of ME/CFS remains unclear, ME/CFS is highly controversial in terms of existence and treatment (Prins et al., 2006), and is a medically unexplained exhaustion. In addition, if ME/CFS lasts longer than six months, the effects caused by the disease are severe enough to result in substantial decline in work, family, social, or school activities (Natelson et al., 2021). COVID-19 is sweeping the world since 2020, studies on COVID-19 have been in hot flood, and through the observation of a large number of clinical cases, patients with long COVID may experience a series of chronic symptoms after COVID recovery, including fatigue, attention problems, and decreased exercise capacity (Nalbandian et al., 2021), which have similarities with ME/CFS (Wong and Weitzer, 2021). Among them, a prospective observational cohort study conducted in 2022 in Germany found that many patients with post-COVID-19 syndrome exhibited symptoms of chronic fatigue syndrome (Kedor et al., 2022), so ME/CFS also attracted the attention of scholars (Groff et al., 2021).

According to the study data of the National Institute for health and care excellence (NICE), approximately 0.2 to 0.4% of the world’s population has suffering from ME/CFS, NICE (2021). Another meta-analysis showed a high global prevalence of 0.8 and 3.3% based on clinical assessment and self-assessment, respectively (Johnston and Brenu, 2013). Affected by COVID-19 epidemic, the incidence of ME/CFS is increasing year by year, which has become an important public health problem worldwide (Lim et al., 2020). And because ME/CFS is a neglected and severe debilitating disease with no proven diagnostic markers and specific therapies (BME/CFS, 2015; Lim et al., 2020), the treatment costs of ME/CFS are around 50% higher than multiple sclerosis (MS) or SLE (Valdez et al., 2019). The majority of ME/CFS patients develop gastrointestinal symptoms, and they have very frequent comorbidities with irritable bowel syndrome (IBS) (Aaron et al., 2000) and inflammatory bowel disease (IBD) (Tsai et al., 2019), it is a reason for the increasing interest in the role of the microbiome in ME/CFS. the role of microbiome in the early development stage when the human immune system continues to play a role is combined with the relevance of the immune system in the pathogenesis and etiology of encephalomyelitis/chronic fatigue syndrome, which can support the view that the composition of intestinal microbiota is closely related to encephalomyelitis/CFS (Bansal et al., 2012; Renz et al., 2012), but whether the gut microbiome is causative in the pathogenesis of ME/CFS remains to be verified. Because the number of ME/CFS patients is increasing worldwide, verifying the relationship between the gut microbiome and ME/CFS is crucial for disease etiology and pathophysiological mechanistic studies. This article focuses on providing evidence for a link between the gut microbiome and ME/CFS.

We performed a two-sample mendelian randomization analysis to examine whether the gut microbiota is causally related to ME/CFS in order to find a basis for potential diagnostic or intervention approaches for ME/CFS. The MR approach eliminated the influence of reverse causality and confounding factors, and finally, we found a link between the gut microbiota and ME/CFS.

## 2. Materials and methods

Mendelian randomization is an important method of causality inference in epidemiology that could circumvent the limitations of traditional observational epidemiology and eliminate a multitude of confounding factors (Emdin et al., 2017). Genetic variants were adopted as instrumental variables (IVs) in this mendelian randomization study to detect causal effects of the exposure on the outcome. Compared with traditional observational epidemiological studies, mendelian randomization is associated with better extrapolation of findings and higher data acquisition (Davey Smith and Ebrahim, 2003). Therefore, in this study, the genome wide association study (GWAS) pooled data was used in a two-sample MR approach to examine whether there is a causal relationship between gut microbiota and ME/CFS. Before sensitivity analysis was performed to ensure the reliability of the results, causal association analysis was conducted by two-sample MR (TSMR) analysis approach.

### 2.1. The assumptions and study design of MR

Summary data for GM and ME/CFS were obtained from GWAS. In conducting a two-sample MR study analysis, we selected single nucleotide polymorphisms (SNPs) that were significantly associated with gut microbiota taxa as instrumental variables based on strict inclusion and exclusion criteria, with gut microbiota as the exposure and ME/CFS as the outcome, and performed a series of sensitivity analyses for the significant associations.

The genetic IVs we chose to conduct TSMR should fulfill the following assumptions (Davey Smith and Hemani, 2014): (1) The genetic variants finally included as IVS must be associated with GM taxa (exposure), (2) the IVS included for use must not be associated with any confounders, and both are independent of each other. And (3) genetic variants must affect ME/CFS only through the gut microbiota and not through any other pathway, i.e., there is no horizontal pleiotropic effect between instrumental variables and outcomes. In parallel, we report our findings based on the MR-STROBE guidelines (Skrivankova et al., 2021).

### 2.2. Data sources and processing

Genome wide association study (GWAS) summary level statistics for gut microbiota and ME/CFS were derived from previous studies or consortia. Genetic instruments for the gut microbiome were selected from single nucleotide polymorphisms (SNPs) associated with the composition of the human gut microbiome, data were derived from a multi-ethnic large-scale GWAS pooled data of 18,437 individuals, 25 cohorts were included from populations of European ethnicity in 11 countries (Kurilshikov et al., 2021). Two hundred eleven taxa (9 phyla, 16 classes, 20 orders, 35 families, and 131 genera) were finally identified, yielding a total of 122,110 variant sites. Meanwhile, we included a study conducted by UK Biobank (Non-cancer illness code, self-reported: chronic fatigue syndrome, 2018), from which ME/CFS data were selected, containing 462,933 individuals (ncase = 2076, ncontrol = 460,857). ME/CFS is confirmed by the patient’s self-reported diagnosis. We used SNPs as instrumental variables, gut microbiota as the exposure variable, and ME/CFS as the outcome variable. Each GWAS involved in this study received ethical approval from the respective institutions. Summary statistics of the included studies of GM are publicly available (www.mibiogen.org website), data of the included studies of ME/CFS are available at: https://gwas.mrcieu.ac.uk/datasets/ukb-b-8961/.

To ensure the authenticity and accuracy of the conclusions on the causal relationship between the gut microbiome and ME/CFS risk and the robustness and reliability of the data, the following quality control steps need to be taken when selecting the best instrumental variables. First, SNPs significantly associated with gut microbiome taxa were selected as instrumental variables at a more comprehensive significance threshold (*p* < 1.0 × 10^−5^). Second, one condition that the MR assumption needs to be satisfied is that there is no linkage disequilibrium (LD) between the instrumental variables, i.e., their distance cutoff is 10,000 kb and the correlation index *R*^2^ ≤ 0.001. Third, palindromic SNPs need to be excluded from instrumental variables to ensure that alleles do not contribute to the outcome between GM taxa and ME/CFS. The F statistical value of SNPs included in this study is greater than 10, indicating that there is no weak instrumental variable bias.

### 2.3. Statistical analysis

All statistical analyses were carried out using R (version 4.1.2). To perform the Mendelian Randomization (MR) analysis, we utilized several methods, including inverse variance weighting (IVW), weighted median, MR-Egger, weighted mode, and simple mode methods. These methods were performed using the “TwoSampleMR” package (version 0.5.6). The MR-PRESSO Global test, which is used to identify and correct for outliers, was carried out using the “MR-PRESSO” package (version 1.0). *p* < 0.05 was considered statistically significant for evidence of a potential causal effect (Waters and Ley, 2019; Xiang et al., 2021).

As the IVW method provides the most precise estimates of causality, it was used as the primary analysis method in this MR study (Burgess et al., 2013; Wang et al., 2021). The IVW method combines ratio estimates with an inverse variance weighted meta-analysis, ensuring that each instrumental variable (IV) is individually valid, with SNP heterogeneity adjustments (Burgess et al., 2013; Bowden et al., 2017). We used the Wald ratio (WR) method to assess the effect of individual IV on causality. In the absence of horizontal pleiotropy, the IVW method is the primary method for estimating causal effect values, enabling unbiased estimates to be obtained without the influence of confounders (Xiang et al., 2021). The presence of heterogeneity determines the selection of a fixed or random-effects model for IVW analysis methods (Liu et al., 2022).

The MR-Egger method included the intercept term in its weighted regression, while the IVW regression did not (Burgess et al., 2017). The intercept term is used by MR-Egger to assess horizontal pleiotropy, which is present when the intercept is not 0. However, the use of the intercept term in MR-Egger regression leads to less statistically efficient estimates of causal effects (Bowden et al., 2015). In contrast, the weighted median approach (WME) provides accurate and consistent estimates of causal effects, even when more than 50% of instrumental variables are invalid (Hartwig et al., 2017). Compared to MR-Egger, WME has important advantages in terms of result accuracy and maintaining more precise causal effect estimates (Bowden et al., 2016; Xiang et al., 2021). Additionally, two other methods were used in MR analysis, including weighted mode (WM) and simple mode (Hartwig et al., 2017; Wu et al., 2020).

#### 2.3.1. Sensitivity analyses

To guarantee the reliability and robustness of the causality assessment results, we also performed sensitivity analyses. Statistics by Cochrane’s *Q*-test to quantify heterogeneity among selected SNPs associated with each bacterial taxa. *p* < 0.5 indicated a clear difference between IVS, indicating heterogeneity. MR-Egger regression was used to test whether there was horizontal pleiotropy among the included SNPs. We also performed the weighted median analysis that was more robust to individual genetic variants with strong outlier causality estimates. Similarly, we implemented the MR-PRESSO test to ensure the accuracy of the results and removed outlier SNPs to correct for horizontal pleiotropic effects (Verbanck et al., 2018).

## 3. Results

### 3.1. Selection of IVs related to GM

After applying quality control steps that removed instrumental variables with LD effect and palindromic SNPs, we identified 1,480 IVs related to gut microbiota, with a significance threshold of *p* < 1.0 × 10^−5^. It includes five biological classifications: phylum, class, order, family and genus, and a total of 211 bacterial taxa. Based on the significance level of *p* < 1.0 × 10^−5^, we identified 63, 114, 144, 262, and 897 SNPs related to gut microbiota in 9 phyla, 16 classes, 20 orders, 35 families, and 131 genera, respectively. Each SNP shows sufficient validity (range between 17.68 and 88.43, all *F* > 10).

### 3.2. MR analysis

**Figure 1:**
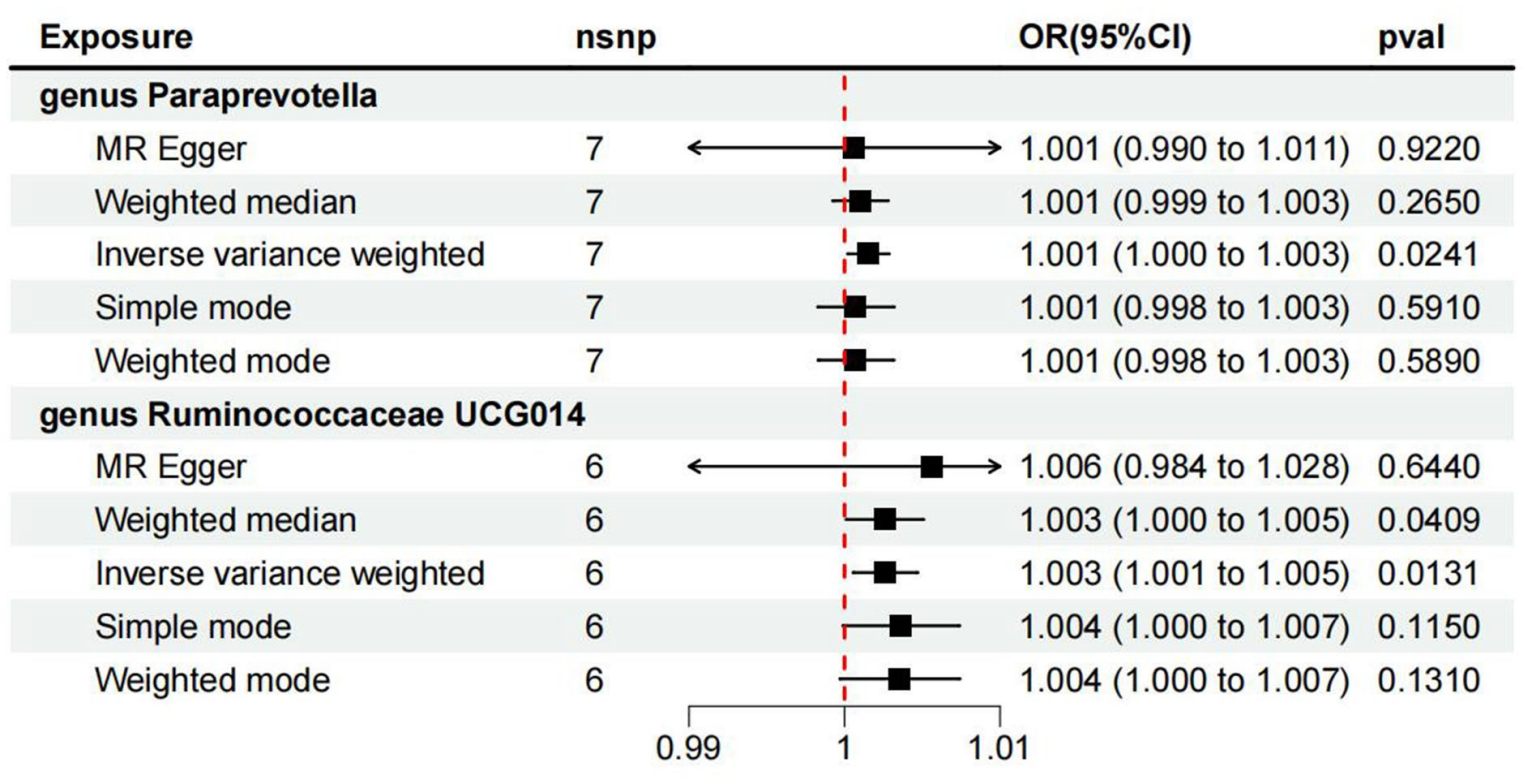
The results of full MR analysis results of five methods.

We found that genus *Paraprevotella* was positively correlated with ME/CFS, indicating that genus *Paraprevotella* was a risk factor for ME/CFS (IVW: OR 1.001, 95% CI: 1.000 to 1.003; *p* value<0.05; Figure 1). However, MR-Egger does not support this result (OR: 1.001, 95% CI: 0.0990 to 1.011; *p* value>0.05), and the analysis result shows that there is no directional level pleiotropy (Egger intercept 0.0001, *p* value = 0.80). MR-PRESSO analysis showed no evidence of horizontal pleiotropy (*p* = 0.81). Cochran’s *Q* test showed no evidence of heterogeneity (Cochran *Q* = 2.98, *p* = 0.81).

For *Ruminococcaceae_UCG_014*, the IVW results showed that the higher level of *Ruminococcaceae_UCG_014* was associated with the higher risk of ME/CFS (OR: 1.003, 95% CI: 1.000 to 1.005; *p* < 0.05; Figure 1). At the same time, WM (Weighted Median) also supports this causal relationship (OR: 1.003, 95% CI: 1.000 to 1.005; *p* value<0.05). However, MR-Egger analysis has no evidence to support this result (OR: 1.006, 95% CI: 0.0984 to 1.028; *p* value>0.05). The details of full MR analysis are shown in Supplementary Table S1, S2. The results of reverse MR analysis is shown in Supplementary Table S1, S2. Therefore, this MR study found that genus *Paraprevotella* and genus *Ruminococcaceae_UCG_014* were positively correlated with the risk of ME/CFS and were risk factors in the pathogenesis of ME/CFS.

### 3.3. Sensitivity analysis

We conducted a series of sensitivity analysis to ensure the reliability and robustness of the research results. We used MR-Egger regression to evaluate the horizontal pleiotropy between the instrumental variables (genetic variants) and the results, and the results showed that there was no evidence of horizontal pleiotropy (all intercepts were *p* > 0.05). In addition, the results of Cochran *Q* test showed that SNPs had no significant heterogeneity (*p* > 0.05). Similarly, the results of MR-PRESSO were not significant, indicating that there was no horizontal pleiotropy (Table 1).

**Table 1:** Results of MR-PRESSO, MR pleiotropy test, and heterogeneity test.

Exposure	Heterogeneity				Pleiotropy		MR-PRESSO	
	MR Egger		IVW		MR Egger		Global test	
	Cochran’s *Q*	*p*-value	Cochran’s *Q*	*p*-value	Egger intercept	*p*-value	RSSobs	*p*-value
Genus *Paraprevotella*	2.949	0.708	2.978	0.812	0.000	0.869	4.074	0.810
Genus *Ruminococcaceae_UCG_014*	2.459	0.652	2.531	0.772	0.000	0.801	3.675	0.804

## 4. Discussion

In the current study, we adopt MR analysis to evaluate the potential causal relationship between gut microbiota and ME/CFS. The results showed that the levels of genus *Paraprevotella* and genus *Ruminococcaceae_UCG_014* were positively correlated with the risk of ME/CFS, that is, genus *Paraprevotella* and genus *Ruminococcaceae_UCG_014* were risk factors of ME/CFS.

Under the unprecedented impact of COVID-19 pandemic, ME/CFS has become a common disease, but it is often ignored in scientific research and health education. Some studies have shown that due to the lack of relevant medical education, about 80% of ME/CFS patients have not received correct and timely diagnosis (Bested and Marshall, 2015; Palacios et al., 2017), which is a major challenge for patients, society and public health system (Jason et al., 2008; ME/CFS, NICE, 2021). At present, the underlying etiology and clear pathophysiological mechanism of ME/CFS are still unclear, but the patients with ME/CFS do have neurological, immune, infectious, muscular, and endocrine pathophysiological abnormalities (König et al., 2022). Patients with ME/CFS often have gastrointestinal symptoms such as diarrhea, constipation and intestinal discomfort (Bested and Marshall, 2015). At the same time, the common comorbidity rate of ME/CFS and irritable bowel syndrome (IBS) is 38% (Chu et al., 2019) to 42% (Nagy-Szakal et al., 2017). When gut microbiome imbalance occurs, it will lead to metabolic, immune and neuro-related diseases (Frémont et al., 2013; Maier et al., 2018; Kashi et al., 2019). Therefore, we consider whether intestinal microorganisms participate in the pathogenesis of ME/CFS.

Phyla *Firmicutes* and *Bacteroidetes* constitute the majority of human intestinal colonies (about 90%), and their proportion is relatively stable and related to the health of host and intestinal metabolism. The two indicators of gene abundance and taxonomic diversity of the intestinal ecosystem are closely related to intestinal health, and both decrease with the increase of age (Bäckhed et al., 2015; Tamburini et al., 2016; Rinninella et al., 2019). Some ME/CFS studies (Sheedy et al., 2009; Frémont et al., 2013; Giloteaux et al., 2016; Armstrong et al., 2017; Nagy-Szakal et al., 2017) found changes and disorders of intestinal microflora, but did not show consistent microbial characteristics (Giloteaux et al., 2016; Navaneetharaja et al., 2016). The *Ruminococcaceae_UCG_014* selected in this MR study belongs to the phyla *Firmicutes*, *Clostridia*, *Clostridiales*, *Lachnospiraceae* and *ruminococcus*; genus *Paraprevotella* belongs to *Bacteroidetes*, *Bacteroidia*, *Bacteroides*, *Prevotellaceae*, and genus *Paraprevotella*. The summary of ME/CFS microbiota research shows that the results of gut microbiota research are not consistent when the exact relationship between intestinal bacterial changes and disease mechanisms cannot be established (Du Preez et al., 2018). Newberry et al. reported in their systematic literature review that there are consistent and conflicting results in ME/CFS microbiome studies, but the overall evidence indicates the existence of biological disorders (Newberry et al., 2018). According to the research report, the number of anaerobic microorganisms in ME/CFS patients will increase (Sheedy et al., 2009), while genus *Ruminococcaceae_UCG_014* and genus *Paraprevotella* are anaerobic bacteria, which are positively correlated with the risk of ME/CFS disease, which is also consistent with our research results.

According to the macrogenomic analysis carried out by Nagy Szakal et al. specific bacterial groups such as *Thickwallida* and *Faecalibacterium* are related to ME/CFS (Nagy-Szakal et al., 2017). In addition, a microbiome study including ME/CFS patients, acute Q fever patients suffering from fatigue and healthy control group found that, when comparing the ME/CFS patients (*n* = 50) with the healthy control group (*n* = 72), the abundance of Phyla *Firmicutes* and *Actinobacteria* increased significantly (Raijmakers et al., 2020). With regard to the genus *Paraprevotella*, *Bacteroidia*, and *Bacteroides* it belongs to have a negative impact on Tourette syndrome (TS). Therefore, we assume that genus *Paraprevotella* may also participate in the neurocognitive mechanism and behavioral performance of patients through the “gut-brain axis,” thus affecting the special symptom of fatigue. Therefore, we believe that the change and imbalance of gut microbiome caused by genus *Ruminococcaceae_UCG_014* and genus *Paraprevotella* may play an important role in ME/CFS, because the increase of intestinal permeability will lead to bacterial translocation, and directly or indirectly affect various cells, their mitochondria and local and systemic immune status through fermentation and metabolites. However, the role of genus *Ruminococca- ceae_UCG_014* and genus *Paraprevotella* in the pathogenesis of ME/CFS is still unclear, and further research is still needed.

The previous review on ME/CFS (Du Preez et al., 2018; Newberry et al., 2018) described the findings of these biological disorders in ME/CFS, which are partially inconsistent but obvious, but their exact role in the pathogenesis of disease is still unclear. This study provides some evidence for the study of pathogenesis and etiology of ME/CFS. The two selected bacteria can also be used as potential biomarkers of disease, providing new perspectives for the follow-up study of disease etiology and pathophysiological mechanism.

This study has the following advantages. First, MR study is more reliable and persuasive than observational study, because gut microbiome is easily affected by diet, some environmental exposure, and other factors, while MR study can reduce the interference of these confounding factors. Secondly, we included the latest large GWAS summary data, and obtained genetic data from large sample populations for analysis. The research results are more reliable than small randomized controlled trials (RCTs). However, it should be noted that this study has certain limitations: First, the MR study cannot determine whether there is data overlap in the included GWAS summary data. Of course, we have reduced the deviation of participants’ overlap to the minimum (Pierce and Burgess, 2013) through F statistics (*F* > 10). Second, due to the lack of initial socio-demographic research data (such as gender and race), further subgroup analysis cannot be carried out. Most of the participants in the GWAS dataset we included are of European origin, so the extrapolation of MR analysis results may be limited. Third, the correction of multiple tests is too conservative and strict. Considering the biological rationality and multi-stage statistical process, we did not conduct multiple tests.

In conclusion, this MR study shows that there is a positive causal relationship between ME/CFS and intestinal microflora, including genus *Paraprevotella*, genus *Ruminococcaceae_UCG_014*. These two bacterial strain are risk factors for ME/CFS and may become new biomarkers of disease, which may provide new insights for the prevention, mitigation and treatment of ME/CFS.

## Data availability statement

The original contributions presented in the study are included in the article/ Supplementary material, further inquiries can be directed to the corresponding author.

## Author contributions

YC and GH performed the computations; GH and WX were involved in the acquisition and interpretation of the data. GH, HM and SG wrote the original manuscript. GH, HM, SG, DW, HW and YC revised the manuscript according to the reviewers’ opinions. All authors contributed to the article and approved the submitted manuscript.

## Conflict of interest

The authors declare that the research was conducted in the absence of any commercial or financial relationships that could be construed as a potential conflict of interest.

## Publisher’s note

All claims expressed in this article are solely those of the authors and do not necessarily represent those of their affiliated organizations, or those of the publisher, the editors and the reviewers. Any product that may be evaluated in this article, or claim that may be made by its manufacturer, is not guaranteed or endorsed by the publisher.

## Supplementary material

The Supplementary material for this article can be found online at: https://www.frontiersin.org/articles/10.3389/fmicb.2023.1190894/full#supplementary-material
